# Targeting the NLRP3 inflammasome in immune cells: A new frontier in autoimmune disease research

**DOI:** 10.1016/j.jtauto.2026.100410

**Published:** 2026-10-22

**Authors:** Aysan Asefpour, Shiva Alipour, Sepideh Sohrabi, Yasser Bagheri, Leili Aghebati-Maleki

**Affiliations:** a Immunology Research Center, Tabriz University of Medical Sciences, Tabriz, Iran; b Department of Immunology, Faculty of Medicine, Golestan University of Medical Sciences, Gorgan, Iran; c Department of Immunology, Faculty of Medicine, Tabriz University of Medical Sciences, Tabriz, Iran; d Student Research Committee, Tabriz University of Medical Sciences, Tabriz, Iran

**Keywords:** NLRP3, Inflammasome, Autoimmune disorders, Innate immunity, Adaptive immunity

## Abstract

Aberrant immune cell activation contributes to autoimmune onset and progression, where emerging evidence demonstrates a critical role for NLRP3 inflammasome activation in this context. NOD-, LRR-, and pyrin domain-containing protein 3 (NLRP3) is a well-known innate pattern recognition receptor (PRR) that is also expressed in adaptive immune cells. Two steps are required for full inflammasome activation: priming and activation. Following NLRP3 activation, caspase-1 cleaves pro-IL-1β and pro-IL-18, leading to their secretion, creating a pro-inflammatory milieu that can enhance the onset and progression of autoimmune diseases. Moreover, NLRP3 activation drives immune cells toward an inflammatory phenotype. Discovering the precise role of the NLRP3 inflammasome in immune cells opens a new avenue for treating autoimmune diseases. This review highlights the function of the NLRP3 inflammasome in innate and adaptive immune cells in the context of autoimmune disorders and discusses therapeutic targeting strategies, emphasizing areas for future research.

## 1 Introduction

Autoimmune diseases are complex conditions in which aberrant activation of innate and adaptive immune cells leads to a failure of self-tolerance, triggering the onset of autoimmune disorders [1], [2]. Innate immune cells initiate immune responses to pathogens or tissue damage, thereby priming the activation of adaptive immune cells [3]. Innate immune cells express various types of pattern recognition receptors (PRRs) that enable them to recognize a wide range of damage-associated molecular patterns (DAMPs) and pathogen-associated molecular patterns (PAMPs) [4]. Nucleotide-binding and oligomerization domain (NOD)-like receptors (NLRs) are cytosolic PRRs that consist of a central nucleotide-binding domain (NACHT) that mediates self-oligomerization; C-terminal leucine-rich repeats (LRRs) that participate in sensing binding ligands; and a variable N-terminal signaling domain (caspase recruitment domain (CARD), pyrin domain (PYD), acidic transactivating domain, or baculovirus inhibitor repeat (BIR)) [5].

Inflammasomes are multiple cytosolic protein complexes that result from cytosolic PRRs activation [6]. There are two pathways for inflammasome activation: the canonical pathway and the noncanonical pathway [7]. Noncanonical inflammasome forms in response to cytosolic LPS, including procaspase-11 in mice and procaspase-4 and -5 in humans [8]. Canonical inflammasome components consist of the nucleotide-binding domain and leucine-rich repeat receptors (NLRs) or AIM2-like receptors (ALRs), an apoptosis-associated speck-like protein containing a CARD (ASC) adaptor protein, and a procaspase-1 effector enzyme [9] (Fig. 1). Activation of pro-caspase-1 generates active caspase-1, which cleaves pro-interleukin-1β (pro-IL-1β) and pro-interleukin-18 (pro-IL-18) into mature IL-1β and IL-18 [10]. Active caspase-1 also cleaves gasdermin D (GSDMD), releasing the GSDMD N-terminal fragment, which induces pyroptosis by forming a pore in the cell membrane. GSDMD-induced pyroptosis then results in the release of IL-1β and IL-18 from the cell [11].

**Fig. 1:**
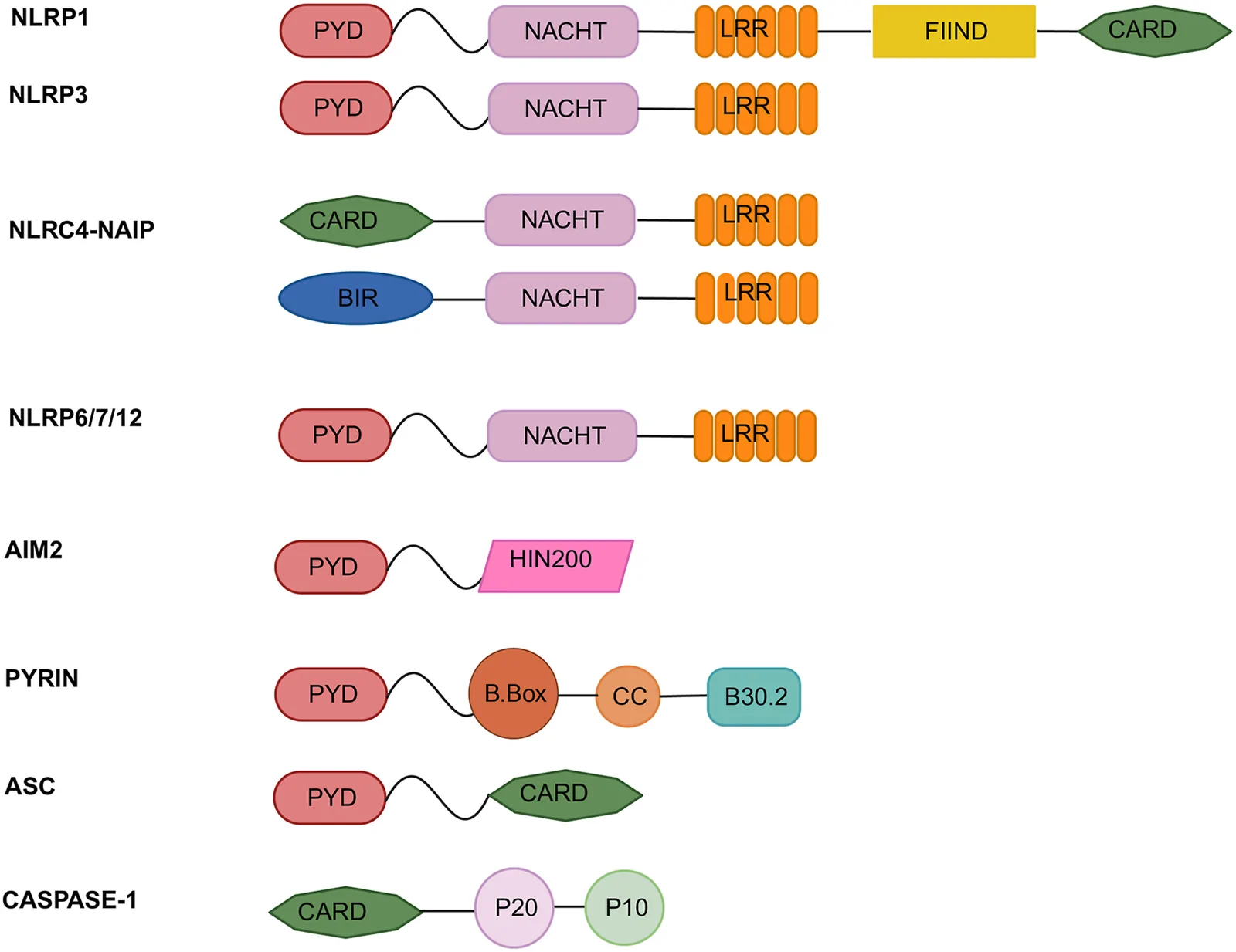
Inflammasome components and domain structure. The oligomerization of several components forms inflammasome complexes. A typical inflammasome consists of three parts: a sensor molecule, the adaptor protein ASC, and the effector molecule pro-caspase-1. The sensors include NLR (NLRP1/3/6/7/12, NLRC4), AIM2, and Pyrin. (AIM2: Absent in melanoma 2; ASC: Apoptosis-associated speck-like protein containing a CARD; B.BOX: B-box domain-containing protein; BIR: Baculovirus inhibitor of apoptosis repeat domain; CARD: Caspase activation and recruitment domain; cc: Coiled-coil; FIIND: Function-to-find domain; HIN200: Hematopoietic interferon-inducible nuclear protein family 200; LRR: Leucine-rich repeat; NAIP: NLR family apoptosis inhibitory protein; NACHT: Nucleoside triphosphatase domain; NLR: Nucleotide-binding and oligomerization domain-like receptors; P10: Caspase-1 p10 subunit; P20: Caspase-1 p20 subunit; P30.2: Caspase-1 p30.2 subunit; PYD: Pyrin domain. Created in Biorender.com).

The first inflammasome identified, NLRP1, possesses an atypical CARD domain at the C-terminus. Another distinctive characteristic is an autoprocessing domain known as the function-to-find domain (FIIND) [12], which generates an inactive, noncovalently linked complex of N-terminal and C-terminal fragments. Upon a triggering signal, the proteasome degrades the N-terminal fragment, releasing the active UPA-CARD fragment to form the inflammasome [13]. Another inflammasome sensor, NLRP6, plays various context-dependent roles, such as regulating the gut microbiota. In NLRP6−/− mice, the gut microbiota induces chemokine (C-C motif) ligand 5, recruits immune cells, and triggers inflammation [14]. In contrast, NLRP6 expression is increased in the lungs of patients with pneumonia [15]. Different bacterial components stimulate various NLRs. For instance, the NLRP7 inflammasome assembles in response to bacterial lipopeptide in human macrophages [16], while *Yersinia* activates the NLRP12 inflammasome, leading to the secretion of IL-1β and IL-18 [17]. Additionally, NLRP12 modulates the gut microbiota: NLRP12−/− mice exhibit increased obesity, reduced glucose tolerance, and heightened inflammation compared to controls [18]. Furthermore, NLRP12 enhances dendritic cell (DC) migration from the periphery to the draining lymph nodes, and its deficiency impairs neutrophil and DC responses to chemokines [19]. Another sensor, NLRC4, has a CARD domain at its N-terminus and requires neuronal apoptosis inhibitor proteins (NAIPs) as sensors for activation in response to bacterial flagellin or the type III secretion system (T3SS) [20]. Due to the CARD domain, NLRC4 can directly activate caspase-1, and NLRC4 also recruits caspase-8 to the inflammasome complex in an ASC-dependent manner [21]. Pyrin, a non-NLR inflammasome sensor with a PYD at the N-terminus, senses the inactivation of Rho GTPase induced by a bacterial toxin, leading to the release of IL-1β and IL-18. Mutations in the pyrin-coding gene are associated with autoinflammatory disorders, such as familial Mediterranean fever (FMF) [22]. Another inflammasome sensor belonging to the ALR family, AIM2, recognizes cytosolic double-stranded DNA (dsDNA) [23]. In addition to its protective role during infection, AIM2 recognition of self-DNA arising from non-infectious inflammation can lead to autoimmune diseases, including Sjögren's syndrome (SS) [24] and psoriasis [25].

Among inflammasomes, NOD-, LRR-, and pyrin domain-containing protein 3 (NLRP3) is the most studied. NLRP3 has a PYD at the N-terminus, which undergoes a homotypic interaction with the PYD of ASC; this interaction ultimately drives assembly of the NLRP3 inflammasome [26]. Activation of the NLRP3 inflammasome requires two signals: (1) a priming signal triggered by activation of TLRs via microbial components, or by tumor necrosis factor (TNF) and IL-1β receptor signaling, and (2) an activating signal resulting from NLRP3's direct or indirect detection of exogenous and endogenous danger signals, such as K+ efflux [27].

The NLRP3 inflammasome plays a crucial role in the pathogenesis of several autoimmune disorders such as rheumatoid arthritis (RA), systemic lupus erythematosus (SLE), multiple sclerosis (MS) [28]. Given the expression of NLRP3 in innate and adaptive immune cells [29] and its role in autoimmune conditions, exploring the NLRP3–autoimmune disease relationship may inform novel therapeutic strategies.

To provide a transparent overview of the available evidence, relevant literature was explored through three major databases: PubMed, Web of Science, and Google Scholar, while ClinicalTrials.gov was queried using the term “NLRP3” to identify registered and ongoing clinical studies relevant to therapeutic targeting of the NLRP3 inflammasome. The literature search was conducted up to August 2026, with an additional focus on identifying the most recent clinical trial results and ongoing therapeutic developments. The search strategy included combinations of the following keywords: “NLRP3,” “innate immunity,” “adaptive immunity,” “autoimmune disease,” “macrophage,” “dendritic cell,” “neutrophil,” “NK cell,” “mast cell,” “MDSCs,” “T helper cell,” “cytotoxic T cell,” “B cell,” “follicular T cell,” “Th1,” “Th2,” “Th17,” “NLRP3 targeting,” and “inflammasome.” Additional disease- and therapy-specific terms were used where appropriate. Literature was mainly selected based on its relevance to the expression, activation, and functional role of NLRP3 in various immune cells, particularly in the context of autoimmune disorders. Literature that discussed only the implication of NLRP3 in autoimmune diseases without specifying the role of an immune cell was excluded. For the therapeutic targeting section, preclinical studies of herbal compounds were excluded. This review follows the general principles of medical review writing [30].

Recent comprehensive reviews have discussed NLRP3 disease by disease in the context of autoimmune conditions, covering its structure and activation mechanisms as well as therapeutic targeting. Specifically, Ren et al. discuss contradictory findings and NLRP3 polymorphisms in autoimmune diseases [31], while Carnazzo et al. describe methods for measuring inflammasome activity and potential biomarkers [32]. This review examines the structure and activation mechanisms of the NLRP3 inflammasome and links them to autoimmune diseases. It adopts an immune cell-centered approach, shedding light on the cell-specific biology of the NLRP3 inflammasome in both innate and adaptive immune cells and on how it modulates their functions in autoimmune disorders. This cell-centered perspective may contribute to the development of novel immune cell-based therapies for autoimmune disorders, such as tolerogenic dendritic cells. Furthermore, this review highlights NLRP3 inflammasome targeting as a promising approach for treating autoimmune diseases and notes ongoing clinical trials, while also discussing the current limitations and challenges of therapeutic NLRP3 inhibition.

## 2 NLRP3 inflammasome: mechanisms required for assembly and activation

NLRP3 is an important PRR expressed in myeloid and lymphoid cells [33], [34], [35]. NLRP3 comprises three domains: the pyrin domain (PYD) at the N-terminus, the central NACHT domain, and the LRR domain at the C-terminus [36]. The PYD undergoes a homotypic interaction with the PYD of ASC [37]. ATP binding to the NACHT domain and its subsequent hydrolysis are essential for NLRP3 self-oligomerization [38]. NLRP3 inflammasome assembly is more complicated than previously shown. Using cryo-electron microscopy, Yu et al. characterized several structural states of human NLRP3 during activation and assembly, supporting a model in which activation proceeds through a transition from a closed cage to an open conformation, with the open NLRP3 octamer representing an intermediate state in oligomerization. This transition is associated with ATP-dependent conformational rearrangements in the NACHT domain. MCC950, a selective NLRP3 inhibitor, binds the NACHT domain at a site distinct from the nucleotide-binding site, stabilizing NLRP3 in its closed conformation and inhibiting ATP hydrolysis. NIMA-related kinase 7 (NEK7) dissociates larger NLRP3 oligomers into NEK7/NLRP3 dimers while preserving the Back-Back interaction. Mutations at several octamer interfaces impair IL-1β signaling and ASC speck formation, with effects varying by mutation and interface [39]. This highlights the potential of the NACHT domain as a therapeutic target in designing direct NLRP3 inhibitors.

The NLRP3 inflammasome is activated via various stimuli, such as bacterial, viral, and fungal pathogens, as well as danger signals [40], [41], [42]. NLRP3 activation requires two steps, priming and activation, as illustrated in Fig. 2 [43]. The priming step, which serves as a license, is triggered by TLR, IL-1β receptor, and TNF receptor signaling, activating the NF-κB transcription factor and increasing NLRP3 expression [44]. The activation step is initiated by various stimuli arising from cellular stress [27]. One such stimulus is activation of the P2X7 receptor (P2RX7) by extracellular ATP [45], [46]. Chen et al. reported that in a chronic dextran sulfate sodium (DSS)-induced inflammatory bowel disease (IBD) mouse model, NEK7 knockdown reduced pyroptosis and the expression levels of NLRP3, caspase-1, and GSDMD-N [47]. ATP binding to P2X7 also triggers the generation of reactive oxygen species (ROS), which subsequently activate downstream PI3K/ERK1/2 pathway [48]. Cytoplasmic Ca^2+^ concentration is another factor in inflammasome activation [49]. The transient receptor potential vanilloid type 1 (TRPV1) channel serves as a route for calcium influx into the cell. It is predominantly expressed in the central nervous system, where it elevates intracellular calcium levels and activates the inflammasome. Zhang et al. reported that in TRPV1 knockout mice with experimental autoimmune encephalomyelitis (EAE), disease symptoms were ameliorated and onset was delayed [50].

**Fig. 2:**
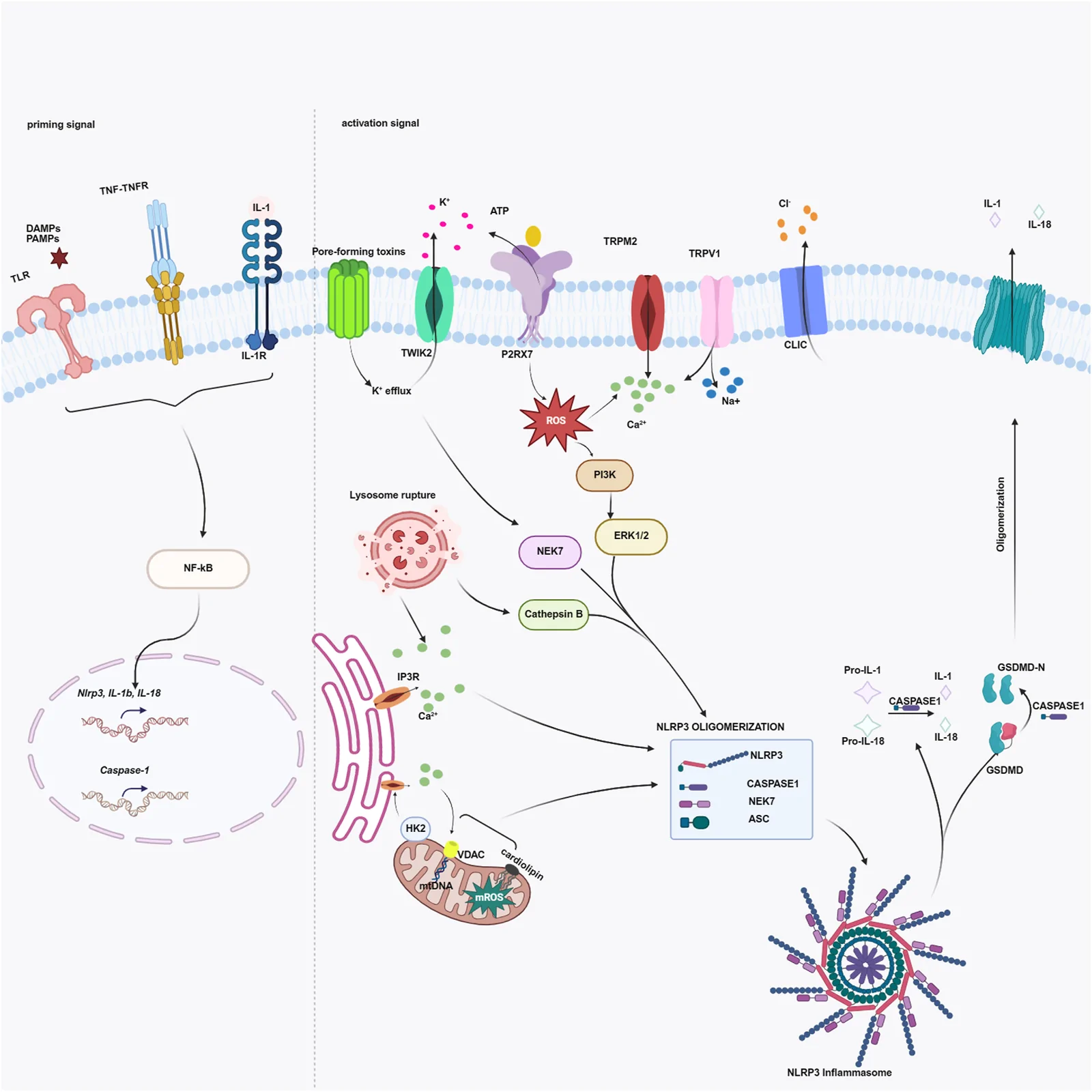
Priming and activation signals required for NLRP3 inflammasome activation. The priming signal results from the activation of TLR, TNFR, and IL1R; subsequently, NF-kB is activated and enhances transcription of NLRP3 inflammasome components and pro-inflammatory cytokines, including IL-1β and IL-18. The activation signal is provided by various stimuli, such as ion efflux, particularly K^+^ efflux, extracellular ATP, lysosome rupture, mitochondrial dysfunction, and ROS generation. Downstream, NLRP3 oligomerizes and leads to caspase-1 activation. Caspase-1 processes pro-IL-1β and pro-IL-18 into IL-1β and IL-18. Also, GSDMD is cleaved by caspase-1, and its N-terminal polymerizes in the cell membrane, creating a pore. (ATP: adenosine triphosphate؛ Ca: calcium؛ Cl: chlorine؛ CLIC: chloride intracellular channel؛ DAMP: danger-associated molecular patterns؛ ERK1/2: extracellular signal-regulated kinase 1/2؛ GSDMD: gasdermin؛ HK2: hexokinase 2؛ IL-1R: interleukin-1 receptor؛ IL-1β: interleukin-1 beta؛ IL-18: interleukin-18؛ IP3R: inositol trisphosphate receptor؛ K: potassium؛ mROS: mitochondrial reactive oxygen species؛ mtDNA: mitochondrial DNA؛ Na: sodium؛ NEK-7: NIMA-related kinase 7؛ NF-κB: nuclear factor kappa light chain enhancer of activated B cells؛ P2RX7: purinergic receptor type 2 family X subunit 7؛ PAMP: pathogen-associated molecular patterns؛ PI3K: phosphoinositide 3-kinase؛ ROS: reactive oxygen species؛ TLR: toll-like receptors؛ TNFR: tumor necrosis factor receptor؛ TRPM2: transient receptor potential melastatin 2؛ TRPV1: transient receptor potential vanilloid type 1؛ TWIK2: two-pore domain weak inwardly rectifying K^+^ channel 2؛ VDAC: voltage-dependent anion channel. Created in Biorender.com).

Cl^−^ efflux also activates the NLRP3 inflammasome. Chloride intracellular channel proteins 1 and 4 (CLIC1 and CLIC4) translocate from the nucleus to the plasma membrane during the LPS priming phase [51]. Release of hexokinase 2 (HK2) from the outer mitochondrial membrane is another trigger of NLRP3 inflammasome activation. It leads to oligomerization of the voltage-dependent anion channel (VDAC) in the same membrane. This oligomerization, together with the release of mitochondrial DNA (mtDNA) into the cytosol, activates the NLRP3 inflammasome [52]. Verma et al. reported that VDAC1 is overexpressed in individuals with IBD, and that treatment with a VDAC1 inhibitor improved outcomes in a mouse model of IBD [53]. Another activation trigger is lysosomal damage. Phagocytosis of endogenous and exogenous crystals disrupts lysosomes and causes leakage of their contents, which the cell recognizes as a danger signal [54], [55].

The NLRP3 inflammasome is activated through three distinct pathways: canonical, noncanonical, and alternative. Canonical activation of the inflammasome is triggered by the various routes described above, leading to caspase-1 activation. Active caspase-1 cleaves GSDMD, releasing its N-terminal fragment (GSDMD-N), which polymerizes in the cell membrane to form a pore and induce pyroptosis [56]. Caspase-1 also mediates maturation of pro-IL-1β, which is ultimately released through the GSDMD-N pore [57]. Analysis of fluid from RA patients demonstrates higher expression and activity of NLRP3, caspase-1, and GSDMD in these patients. This suggests that pyroptosis contributes to joint inflammation [58]. Pyroptosis-mediated pathogenic effects may extend beyond those mediated by inflammasome-derived cytokines. In EAE, GSDMD-dependent pyroptosis is activated in the CNS and contributes to disease pathogenesis. Administration of recombinant IL-1β and IL-18 to GSDMD−/− mice partially exacerbated disease severity but failed to restore the disease progression observed in wild-type mice. This suggests that the pathogenic effects of GSDMD-mediated pyroptosis may rely on other released factors [59].

Caspase-4 (referred to as caspase-11 in mice) and caspase-5 are activated in the noncanonical inflammasome upon detection of cytosolic lipopolysaccharide (LPS) from Gram-negative bacteria [60]. Caspase-11 is activated upon detecting LPS from Gram-negative bacteria, leading to cleavage of GSDMD and subsequent activation of the NLRP3 inflammasome [61]. Concurrent detection of LPS and bacterial mRNA, indicative of live infection, by procaspase-11 is essential for activating the noncanonical inflammasome; procaspase-11 engages the LRR and PYD of NLRP3, thereby activating the NLRP3 inflammasome [62]. Notably, caspase-11 is activated prior to the canonical NLRP3 inflammasome pathway: caspase-11 alters cell membrane permeability, causing a drop in intracellular potassium, which in turn triggers activation of the canonical NLRP3 inflammasome [63].

Unlike the canonical and non-canonical pathways, alternative NLRP3 activation does not require K^+^ efflux. Instead, it is initiated by cell-surface LPS engagement and proceeds through TLR4 signaling involving receptor-interacting serine/threonine-protein kinase 1 (RIPK1), Fas-associated protein with death domain (FADD), and caspase-8 [64].

These cellular stress pathways may activate the NLRP3 inflammasome in the context of autoimmune disease. In RA, ROS generation activates the NLRP3 inflammasome in CD4^+^ T cells and is associated with inflammatory responses [65], whereas in SLE patients, anti-dsDNA enhances mitochondrial ROS levels in macrophages, leading to NLRP3 activation [66]. Also, inhibition of K^+^/Cl^−^ efflux and mitochondrial dysfunction can prevent EAE progression [67]. It is noteworthy that NLRP3 activation mechanisms in immune cells in the context of autoimmune disease extend beyond these general mechanisms and depend on the type of immune cell and disease context.

## 3 The role of NLRP3 in innate immune cells and its contribution to the onset and progression of autoimmune disorders

Autoimmune disorders comprise a diverse array of variables and conditions, with multiple elements contributing to their development. Innate immune cells play an essential role as the first line of defense, execute a crucial function in launching immunological responses and also contribute to the development and progression of autoimmune disorders. A viral infection with antigenic similarity to self-antigens or a strong activation of innate immunity followed by tissue damage can create a platform for initiating autoimmune disease [68]. Innate immune cells are stimulated by the detection of DAMPs and PAMPs, which, as previously mentioned, activate the inflammasome [69]. Excessive inflammasome activation induces inflammation and pathological tissue damage while also stimulating adaptive immunity, providing a basis for the development of autoimmune disorders [28]. Fig. 3 illustrates the role of NLRP3 inflammasome activation in innate immune cells within the context of autoimmunity.

**Fig. 3:**
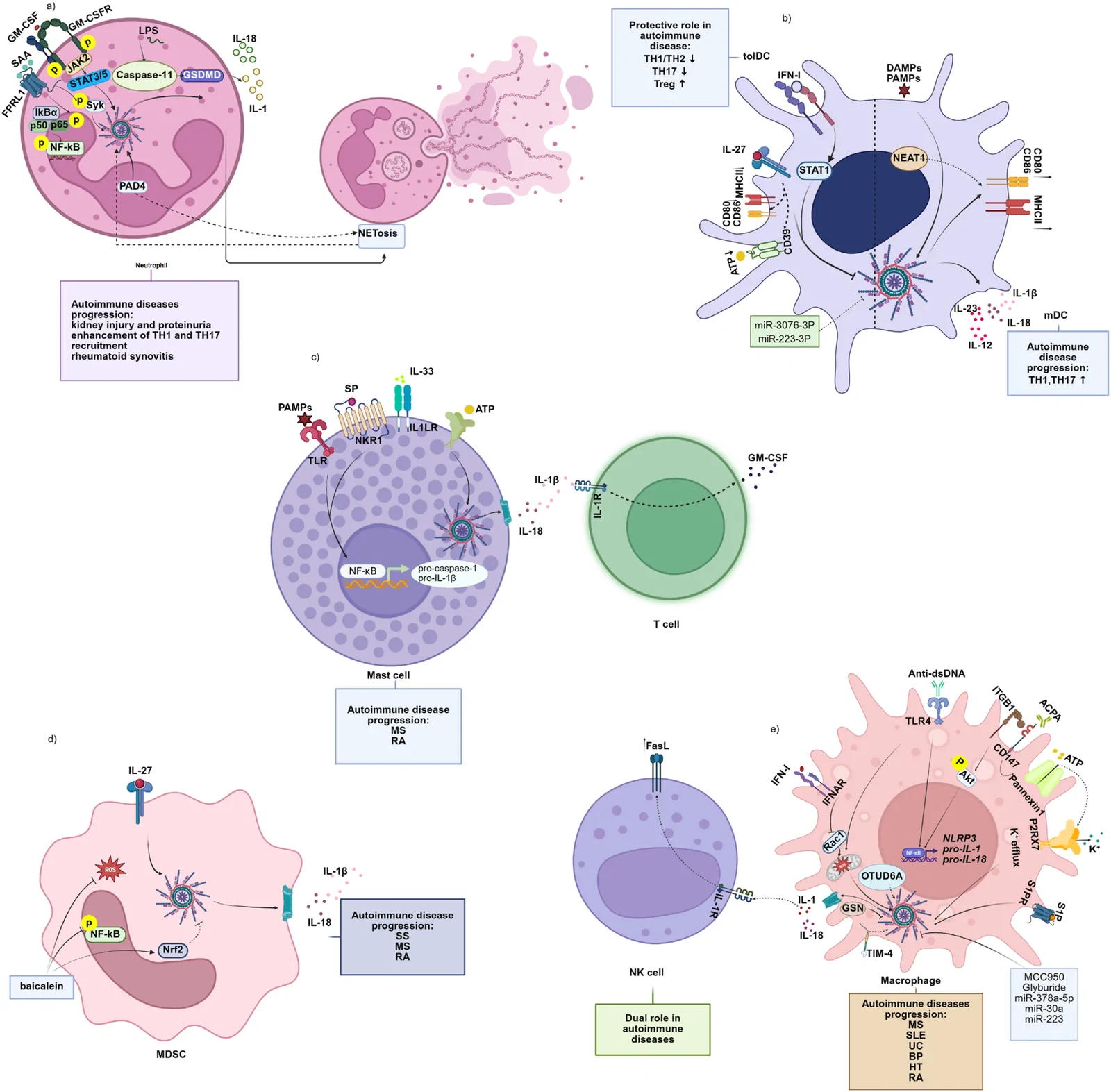
The role of NLRP3 inflammasome activation in innate immune cells in autoimmune disease. a) GM-CSFR activation induces STAT3/5 activation in neutrophils, which activates the NLRP3 inflammasome. PAD4 enhances both NETosis and NLRP3 inflammasome activation. As a result of the NLRP3 inflammasome activation, IL-1β promotes NETosis through a positive feedback loop. b) IL-27 and IFN-I suppress NLRP3 inflammasome activation. Inhibition of the NLRP3 inflammasome reduces the expression of MHC-II and CD80, 86 in tolerogenic dendritic cells (tolDCs). The activation of NEAT1 and the NLRP3 inflammasome enhances the expression of MHC-II and CD80, 86 in mDCs. c) SP, IL-33, and recognition of PAMPs provide the priming signal in mast cells. d) IL-27 activates NLRP3 in myeloid-derived suppressor cells, while baicalein inhibits NF-κB phosphorylation, ROS generation, and activates Nrf2. e) Several stimuli induce NLRP3 inflammasome activation in macrophages. IL-1β secreted by macrophages enhances FasL expression in NK cells. (ACPA: anti-citrullinated protein antibody؛ Akt: protein kinase B؛ Anti-dsDNA: anti-double stranded DNA؛ ATP: adenosine triphosphate؛ BP: Bullous pemphigoid؛ CD39: Ectonucleoside triphosphate diphosphohydrolase-1 also known as Cluster of Differentiation 39؛ DAMPs: damage‐associated molecular patterns؛ FasL: Fas ligand؛ FPRL1: Formylpeptide Receptor Like 1؛ GM-CSF: granulocyte-macrophage colony-stimulating factor؛ GM-CSFR: granulocyte-macrophage colony-stimulating factor receptor؛ GSN: gelsolin؛ HT: Hashimoto's thyroiditis؛ IFN-1: interferon type 1؛ IFNAR: type-I IFN receptor؛ IκB-α: NF-kappa-B inhibitor alpha؛ IL1RL: Interleukin 1 receptor-like 1؛ ITGB1: integrin beta 1؛ JAK2: Janus kinase 2؛ mDC: mature dendritic cell؛ MDSCs: myeloid-derived suppressor cells؛ MHC-II: major histocompatibility complex class II؛ miR: microRNA؛ MS: Multiple sclerosis؛ NEAT1: nuclear paraspeckle assembly transcript 1؛ NETs: neutrophil extracellular traps؛ NF-κB: nuclear factor-kappa B؛ NK1R: neurokinin 1 receptor؛ Nrf2: nuclear factor erythroid 2–related factor 2؛ OTUD6A: ovarian tumor deubiquitinase 6A؛ P2RX7: purinergic receptor type 2 family X subunit 7؛ PAD4: peptidyl arginine deiminase 4؛ PAMPs: pathogen‐associated molecular patterns؛ RA: rheumatoid arthritis؛ Rac1: Ras-related C3 botulinum toxin substrate 1؛ S1P: sphingosine-1-phosphate؛ SAA: serum amyloid A؛ SP: substance P؛ SS: Sjögren's syndrome؛ STAT: signal transducer and activator of transcription؛ SYK: spleen associated tyrosine kinase؛ TIM4: T-cell immunoglobulin and mucin domain-containing 4؛ TLR: toll-like receptor؛ tolDC: tolerogenic dendritic cells؛ UC: ulcerative colitis. Created in Biorender.com).

### 3.1 Neutrophils

Neutrophils are polymorphonuclear (PMN) cells that serve as the immune system's primary defense and, furthermore, function as regulators of adaptive immunity [70]. A unique feature of neutrophils is the release of neutrophil extracellular traps (NETs), which are traps composed of DNA strands, histone proteins, and the enzymes neutrophil elastase and myeloperoxidase [71]. There is a positive correlation between NLRP3 activation and NETosis. NLRP3-dependent IL-1β release not only promotes NETosis but also further stimulates NLRP3 activation in a positive feedback loop [72], [73]. The enzyme peptidyl arginine deiminase 4 (PAD4), found in the nucleus and cytoplasm, decondenses chromatin and increases the levels of NLRP3 and ASC post-transcriptionally. Subsequently, caspase-1 is activated, leading to cleavage of GSDMD, which permeabilizes the nuclear and plasma membranes, resulting in chromatin release and the formation of NETs [74]. Activation of the noncanonical inflammasome pathway by Gram-negative bacterial LPS drives cleavage of GSDMD by active caspase-11, ultimately resulting in NETosis. Caspase-11 is more efficient than caspase-1 at cleaving GSDMD and inducing pyroptosis [75]. Because neutrophils resist pyroptosis during canonical inflammasome activation, they can serve as a sustained source of IL-1β [76]. In addition, NLRP3 inflammasome activation in neutrophils modulates their migration to the inflammatory site, where IL-1β activates endothelial cells and enhances the expression of P-selectin on the endothelial cell surface. This results in elevated neutrophil recruitment to the site of inflammation [77]. In inflammatory situations, serum amyloid A (SAA), an acute-phase protein, triggers the NLRP3 inflammasome within neutrophils by providing both priming and activating signals. SAA enhances the expression of NLRP3 and pro-IL-1β, resulting in the production of mature IL-1β [78].

Neutrophils are involved in the pathogenesis of various autoimmune diseases. For example, alongside T cells, brain-infiltrating neutrophils play a significant role in the complex pathogenesis and progression of EAE; disease severity is reduced in NLRP3 knockout (KO) mice. NLRP3 influences neutrophil function, including NET formation: the chemokine receptors CXCR2 and CXCR4 are associated with NET formation and show reduced expression in NLRP3 KO mice. Accumulation of NETs exacerbates disease by recruiting Th1 and Th17 cells, and NLRP3 further worsens disease severity by promoting NET formation [79].

In mice carrying an NLRP3 gain-of-function mutation (Nlrp3-R258W), infiltration of inflammatory cells, specifically neutrophils, plays a key role in kidney damage and lupus progression [80]. Also, in the context of SLE, NETs, together with the cathelicidin LL-37, a cationic peptide synthesized by neutrophils, activate the inflammasome in macrophages. Macrophage-secreted IL-18 increases NETosis, which establishes a continuous cycle of elevated IL-1β and IL-18, further enhancing inflammation and NET formation [81]. Another stimulator of the NLRP3 inflammasome in neutrophils, granulocyte-macrophage colony-stimulating factor (GM-CSF), increases NLRP3 and IL-1β expression and activation in neutrophils without requiring a priming signal. GM-CSF activates Janus kinase 2 (JAK2) by binding to its cytokine receptor; activated JAK2 phosphorylates the receptor's cytoplasmic tail, initiating the signal transducer and activator of transcription (STAT) 3/5 pathway. This signaling cascade drives NLRP3 expression and IL-1β release. In RA, IL-1β secretion in the synovium contributes to rheumatoid synovitis. Tofacitinib inhibits JAK2, thereby preventing GM-CSF signaling and limiting the activity of synovial T helper cells, which generate GM-CSF and induce IL-1β production from neutrophils [82]. Collectively, NLRP3 inflammasome activation in neutrophils, accompanied by NETosis, can play an important role in situations in which further inflammation contributes to autoimmune conditions like RA and SLE. This suggests that neutrophils can be considered a potential therapeutic target.

### 3.2 Macrophages

Macrophages are essential cells with multifaceted functions in the innate immune system. Blood monocyte-derived macrophages infiltrating inflamed tissue contribute to the pathophysiology of autoimmune disorders. Macrophages are crucial in T cell activation through antigen presentation and cytokine secretion [83]. In response to stimuli, macrophages are polarized towards the classical M1 or alternative M2 phenotype. M1 macrophages with an inflammatory phenotype are activated in response to infection or injury, while M2 macrophages participate in tissue repair, restriction of inflammation, angiogenesis, tumor promotion, and allergic and parasitic responses by secreting anti-inflammatory cytokines [84]. NLRP3-induced M1 polarization fosters inflammation in various conditions. For instance, activation of NLRP3 in microglia, macrophages residing in the CNS, enhances local inflammation. In a cuprizone (CPZ)-induced mouse model of MS, TRPM2 plays a crucial role in regulating NLRP3 and caspase-1 expression [85]. Supporting this, LPS-induced microglial activation led to M1 phenotypic polarization and increased iNOS levels, while treatment with MCC950, a selective NLRP3 inflammasome inhibitor, suppressed M1 polarization. Inhibition of NLRP3 by MCC950 reduced demyelination and disease severity in EAE mice [86]. Surprisingly, conventional interferon-β (IFN-β) therapy for EAE is effective only when the NLRP3 inflammasome is active during disease, since NLRP3 activation promotes disease development. Notably, IL-1β and caspase-1 levels are increased in type I interferon receptor (IFNAR) knockout macrophages compared to wild-type macrophages. Mechanistically, IFNAR downstream signaling inhibits NLRP3 inflammasome activation triggered by ATP-generated ROS [87]. In EAE, ROS accumulation in spinal cord macrophages induces NF-κB activation and increases NLRP3 expression, providing a priming signal for inflammasome activation. Treatment with the recombinant flagellin A protein containing linked C- and N-terminal ends (FLaAN/C) inhibited the ROS/NF-κB/NLRP3 pathway. In treated mice, the peak of EAE was delayed, and pyroptosis-mediated progression was reduced [88].

In addition to MS, NLRP3 inflammasome activation in macrophages has a role in SLE pathogenesis. Anti-dsDNA antibodies trigger the NLRP3 inflammasome in monocyte-derived macrophages, leading to caspase-1 activation and IL-1β release in SLE patients. In detail, anti-dsDNA activates the NLRP3 inflammasome through two mechanisms that deliver both priming and activation signals: enhancing mitochondrial ROS production and binding to TLR4, which subsequently activates the NF-κB pathway [66]. A relationship between lupus nephritis and NLRP3 inflammasome activation has also been observed. Sphingosine-1-phosphate (S1P) levels are increased in the kidneys and serum of lupus-prone MRL/lpr mice. S1P, via its receptor (S1PR), activates the NLRP3 inflammasome in macrophages, resulting in M1 macrophage polarization, which plays an important role in the progression of lupus nephritis [89].

Interleukin-10 (IL-10) suppresses NLRP3 inflammasome activation in colonic macrophages; in IL-10−/− mice, NLRP3 and ASC expression are increased. Notably, NLRP3 inhibition by glyburide reduces colitis [90]. As a novel potential therapeutic approach to treating IBD, hucMSC-derived exosomes downregulate the expression of NLRP3, ASC, caspase-1, IL-1β, and IL-18 in macrophages and delay pyroptosis by inhibiting N-terminal cleavage of GSDMD in a DSS-induced mouse model of colitis. Similar effects are observed in vitro in human THP-1 cells and mouse peritoneal macrophages. hucMSC-derived exosomes contain microRNAs that target NLRP3; miR-378a-5p strongly suppresses NLRP3 expression and downregulates ASC and IL-18 protein levels [91]. Beyond NLRP3 activation pathways, the deubiquitinase enzyme ovarian tumor deubiquitinase 6A (OTUD6A) increases NLRP3 levels by inhibiting its proteasomal degradation. OTUD6A directly interacts with the NACHT domain, thereby removing K48-linked polyubiquitin chains from NLRP3 at K430 and K689. In LPS- and ATP-treated bone marrow-derived macrophages (BMDMs) from Otud6a−/− mice, ASC speck formation and IL-1β release are considerably reduced, leading to improved colitis outcomes [92].

In the context of RA, CD14-positive monocyte/macrophage cells entering the synovium are the principal source of active caspase-1. Inhibition of the NLRP3 inflammasome by MCC950 in a murine model of CIA reduces inflammation and cartilage degradation [93]. Clinically, analysis of RA patients' peripheral blood mononuclear cells (PBMCs) indicates that macrophages promote systemic inflammation in RA. Application of hUCB-MSCs induces macrophage polarization toward the M2 phenotype and suppresses the NLRP3 inflammasome via an IL-1β paracrine loop [94]. Gelsolin (GSN), an actin-binding protein that is significantly reduced in the plasma of RA patients, inhibits NLRP3 in macrophages. The NACHT and PYD of NLRP3 interact with the N-terminus of cytosolic GSN during the priming stage of NLRP3 inflammasome activation upon LPS stimulation. This interaction prevents NLRP3 translocation to mitochondria by interfering with the NLRP3–mitochondrial antiviral-signaling protein (MAVS) interaction, leading to reduced IL-1β secretion and pyroptosis-induced inflammation [95]. IL-1β concentration is elevated in anti-citrullinated protein antibody (ACPA)-positive RA patients compared to ACPA-negative patients. Mechanistically, ACPA stimulates the NLRP3 inflammasome in macrophages by enhancing the interaction between CD147 (which promotes M1 macrophage differentiation) and integrin beta 1 (ITGB1); this process triggers phosphorylation of protein kinase B (PKB, also known as Akt) and activates NF-κB [96]. Furthermore, Cai et al. demonstrate that in the citrullinated collagen-induced arthritis (C-CIA) mouse model of RA, ACPA act as a danger signal and activate the TLR4/NF-κB signaling pathway. Mechanistically, ACPA also provides an activation signal for NLRP3 inflammasome assembly in macrophages by promoting extracellular ATP release via the Panx-1 channel and K+ efflux via the two-pore domain weak inwardly rectifying K+ channel 2 (TWIK2). Inhibition of the ATP/K+ efflux axis decreases arthritis progression and macrophage pyroptosis [97]. Post-transcriptional regulation of NLRP3 by microRNAs reduces joint inflammation and bone damage. TNFα activates the transcription factor RelA via the NF-κB pathway, increasing NLRP3 expression while simultaneously decreasing miR-30a, which inhibits NLRP3 expression in mouse BMDMs by binding to the 3′-untranslated region (3′UTR) of NLRP3 mRNA [98]. Similarly, exosomes derived from bone marrow mesenchymal stem cells (BMSCs) containing miR-223 suppress the NLRP3 inflammasome in macrophages and inhibit IL-1β, IL-18, and TNF-α, thereby decreasing the inflammatory response in a rat model of RA [99].

NLRP3 is also expressed in epidermal keratinocytes and skin-infiltrating PMN cells in patients with bullous pemphigoid (BP), where CD163-positive macrophages are the predominant infiltrating population; NLRP3 expression has also been observed in epithelial cells and fibroblasts within the dermis and blister cavities. Blister fluid (BF) induces priming and activation of the NLRP3 inflammasome in human macrophages. Interleukin-17 (IL-17) and interleukin-23 (IL-23) induce macrophages to produce IL-1β, which further activates the NLRP3 inflammasome in a self-amplifying cycle. IL-1β-activated macrophages initiate inflammation and immune responses by secreting matrix metalloproteinase-9 (MMP-9), which degrades matrix components into peptides that promote disease development [100]. In the context of Hashimoto's thyroiditis (HT), it has been shown in vitro that macrophage activation and polarization toward M1, induced by LPS and interferon-gamma (IFN-γ), elevate the expression of NLRP3 and T-cell immunoglobulin and mucin domain-containing 4 (TIM4). Silencing TIM4 in M1 macrophages markedly decreased NLRP3 expression. Coculture of M1 macrophages with the human thyroid follicular cell line Nthy-ori 3-1 enhanced apoptosis and inflammation in Nthy-ori 3-1 cells. These findings indicate potential therapeutic targets for managing HT, but the precise mechanism underlying the link between these two molecules requires additional research [101]. Taken together, NLRP3-mediated macrophage activation is triggered by various stimuli that are associated with specific autoimmune disorders, including ACPA in RA and anti-dsDNA in SLE, highlighting their crucial role in autoimmunity.

### 3.3 Dendritic cells

Dendritic cells (DCs) uniquely activate naïve T cells as professional antigen-presenting cells. They play a crucial function in the immune system by connecting innate and adaptive immunity. DCs are categorized into two primary types: conventional DCs (cDCs) and plasmacytoid DCs (pDCs) [102].

Classical dendritic cells are categorized into two subtypes: cDC1 and cDC2 [103]. cDC2 is the primary subset that secretes IL-1β in response to extracellular PAMPs after activating the NLRP3 inflammasome. The stimulation of cDC2 with the non-classical stimulus L18-muramyl dipeptide (MDP) results in NLRP3 inflammasome hyperactivation without inducing pyroptosis. In the hyperactivated state, cDC2 enhances the expression of co-stimulatory molecules such as CD40 and CD86, and in addition to secreting IL-1β, it also secretes IL-12 and IL-23, which facilitate the activation of T helper-1 and T helper-17, respectively. Activation of cDC2 with ATP and nigericin induces pyroptosis, resulting in the exclusive release of IL-1β [104]. Bencze et al. found that only the NLRP3 receptor and ASC are detectable at the protein level in resting pDCs. Unlike myeloid lineage cells, only gram-negative pathogenic bacteria may cause the synthesis of pro-IL-1β in pDCs. The production of mature IL-1β requires both priming and activating signals, along with diverse activators that induce different levels of pro-IL-1β expression. Conversely, the synthesis of type 1 interferon by pDCs, the primary source of type 1 IFN, augments the expression of IL-1β transcriptional inhibitor molecules at the mRNA level and NLRP3 inhibitors via STAT1 activation in these cells, consequently suppressing the activation of the NLRP3 inflammasome and the production of IL-1β [105].

Considering the pivotal function of DCs in inducing adaptive immune responses and in maintaining central and peripheral tolerance, the generation of tolerogenic dendritic cells is advantageous for the management of autoimmune disorders [106]. For instance, IL-27 is expressed at higher levels in cDCs than in pDCs and regulates cDC function. IL-27 reduces the expression of CD80, CD40, and MHC-II in dendritic cells, and enhances its own release from dendritic cells through a positive feedback mechanism. IL-27 decreases Th1 and Th17 differentiation by enhancing the expression of anti-inflammatory cytokines and reducing the production of inflammatory cytokines. IL-27 markedly suppresses the NLRP3 inflammasome through ectonucleoside triphosphate diphosphohydrolase-1 (also known as CD39), which decreases extracellular ATP levels. Administration of IL-27-treated tolDCs markedly ameliorates EAE [107].

Nuclear paraspeckle assembly transcript 1 (NEAT1) is a long non-coding RNA located in the nucleus and cytoplasm of dendritic cells. NEAT1 expression is elevated in mature DCs activated by LPS. Knockdown of NEAT1 in DCs decreases expression of the co-stimulatory molecules CD80, CD86, and major histocompatibility complex (MHC) class II in LPS-stimulated DCs. Additionally, NEAT1 knockdown reduces inflammatory cytokines, including IL-1β, IL-6, IL-12, and IL-17A, while increasing anti-inflammatory cytokines such as IL-10 and transforming growth factor beta (TGF-β). NEAT1-knockdown DCs exhibit a tolerogenic phenotype through miR-3076-3p, which markedly reduces the mRNA expression levels of NLRP3, caspase-1, and ASC. These tolerogenic DCs enhance the regulatory T cell population while reducing Th17 cell numbers. NEAT1 knockdown in DCs mitigates experimental autoimmune myocarditis (EAM) induced by subcutaneous myosin injection in BALB/c mice, and also promotes immunological tolerance in a cardiac transplantation model [108]. Similarly, miR-223-3p also promotes the formation of tolDCs. As a crucial regulator of innate immunity during inflammatory responses, miR-223-3p downregulates caspase-1 and IL-1β production by directly interacting with NLRP3 and blocking its activity. Inhibition of NLRP3 in DCs promotes a tolerogenic phenotype, altering their functional properties. Introduction of miR-223-3p-transfected DCs into EAM animals markedly alleviated pericardial inflammation, with an increased number of FOXP3+ cells [109]. As another advantage of tolDCs in autoimmune conditions, tolDCs confer protection in the CIA mouse model by mitigating joint swelling, inflammatory cell infiltration, and cartilage and bone damage. TolDCs also inhibit the NLRP3 inflammasome, thereby reducing pyroptosis and the release of IL-1β and IL-18. Additionally, tolDCs increase the Th2-to-Th1 ratio, elevate Treg levels, and reduce Th17 levels [110]. Furthermore, glyburide inhibits the NLRP3 inflammasome in human monocyte-derived DCs and induces DC polarization toward the tolDC phenotype. Insulin-loaded and unloaded tolDCs both show an increase in regulatory and Th2 phenotypes when cocultured with T cells [111].

Thus, given the DCs' special capacity for adaptive immune activation, NLRP3 inhibition aimed at generating tolDCs may ameliorate autoimmune disease development. However, further investigations are needed to verify its effectiveness in the treatment of autoimmune disorders in humans.

### 3.4 Natural killer cells

NK cells are generated from the lymphoid lineage and play a crucial role in the initial innate immune response against viral infections. NK cells are categorized as cytotoxic CD56^dim^ CD16^+^ in peripheral blood circulation and cytokine- and chemokine-producing CD56^bright^ CD16^−^ in lymphoid tissues [112]. NK cells may play a protective or detrimental role in autoimmune disorders, depending on their phenotype and the environment in which they operate. For example, CNS-resident NK cells contribute to protection against MS by inhibiting myelin-reactive Th17 cells [113]. Conversely, CD27low NK cells (analogous to CD56dim NK cells in humans) represent the predominant population during the pre-onset phase of EAE, with increased cytotoxic capacity. CD27low NK cells promote disease progression by enhancing DC maturation [114].

Exposure to trichloroethene (TCE) promotes the development of autoimmune diseases, including autoimmune hepatitis (AIH) and SLE, in a mouse model. TCE significantly enhances the infiltration of NK cells by activating the NLRP3 inflammasome in hepatic immune cells [115]. In the context of memory-like responses in NK cells, the formation of hapten-induced memory NK cells depends on activating the NLRP3 inflammasome and releasing IL-18 by skin-resident macrophages, which detect stress signals from melanocytes. Hapten-induced memory NK cells are essential for eliciting contact hypersensitivity responses to monobenzone [116]. Given that many studies have shown the dual role of NK cells in autoimmune diseases [117], [118], identifying the role of NLRP3 inflammasome activation in NK cells in autoimmune diseases requires further study.

### 3.5 Mast cells

Mast cells are tissue-resident cells located throughout the body, particularly surrounding blood vessels. In addition to engaging in allergy and helminth responses, mast cells fulfill various functions, including angiogenesis, homeostasis, and immune response modulation [119]. They also participate in the pathophysiology of autoimmune disorders [120]. Mast cells express components of the NLRP3 inflammasome and IL-1β. Studies of the skin urticarial rash of patients with cryopyrin-associated periodic syndrome (CAPS), an autoinflammatory disorder, have shown that mast cells are the primary cells secreting IL-1β in these patients' skin through the NLRP3 inflammasome [34], [121]. Activation of mast cells by substance P and IL-33 leads to the expression of procaspase-1 and pro-IL-1β and the release of IL-1β [122]. Also, estrogen activates the NLRP3 inflammasome in mast cells, promoting the progression of endometriosis [123].

In terms of autoimmune diseases, Russi et al. revealed that myelin oligodendrocyte glycoprotein (MOG)-specific Th cells accumulate in the meninges of mice with early EAE. This indicates that these MOG-specific Th cells have previously encountered MOG-presenting antigen-presenting cells (APCs) in the meninges. The meninges are recognized as a locus for immunological responses in both EAE and MS. Mast cells attract T lymphocytes to the meninges by the secretion of chemokines. As a principal source of IL-1β release, mast cells stimulate T cells to produce GM-CSF, hence enhancing the recruitment of inflammatory monocytes [124]. Also, mast cells contribute to the pathophysiology of RA by releasing IL-1β [125]. Collectively, mast cells serve as a source of IL-1β in tissues, especially in the meninges, where it stimulates T cells to secrete cytokines like GM-CSF, contributing to further inflammation and promoting autoimmune responses. However, further investigations are needed to clarify mast cells' contribution to autoimmune conditions, given that IL-1β-derived inflammation can promote disease progression.

### 3.6 Myeloid-derived suppressor cells (MDSCs)

Myeloid-derived suppressor cells (MDSCs) are a heterogeneous collection of immature myeloid cells that possess inhibitory functions and facilitate tumor advancement [126]. MDSCs also play a role in autoimmune disorders [127]. For instance, in Sjögren's syndrome (SS), MDSCs with elevated NLRP3 inflammasome activity secrete more IL-18 and IL-1β. Notably, IL-27 activates the NLRP3 inflammasome in MDSCs, whereas this activation is suppressed in IL-27 −/− mice [128]. In the spleen of the CIA model, the number of MDSCs is increased relative to the control group, and these cells produce elevated levels of TNF-α and IL-1β, thereby promoting Th17 differentiation. Moreover, MDSCs are elevated in the serum of patients with RA, where they contribute to disease pathophysiology by secreting IL-1β and enhancing Th17 levels [129]. Furthermore, CD11b + Gr-1+ mice MDSCs play a role in the pathogenesis of EAE by secreting IL-1β and promoting T-cell differentiation into Th17 cells [130]. In a study investigating the role of MDSCs in the pathogenesis of pristane-induced lupus nephritis (LN) mouse model, baicalein, a flavonoid, has been shown to prevent MDSC expansion and alleviate symptoms by reducing NLRP3 inflammasome activation and the levels of ROS or NF-κB phosphorylation, as well as increasing the activity of nuclear factor E2-related factor 2 (Nrf2), which possesses antioxidant properties [131]. Thus, MDSC-derived IL-1β plays an important role in autoimmune disease progression by inducing the polarization of Th cells into Th17 cells.

## 4 The role of NLRP3 in adaptive immune cells and its contribution to the onset and progression of autoimmune disorders

The adaptive immune system plays a central role in autoimmune diseases, with the breakdown of self-tolerance being a primary reason for their genesis and progression [132]. NLRP3, while recognized as a receptor in innate immune cells, has been demonstrated to influence autoimmune disorders through its activation in adaptive immune cells [29]. IL-1β and IL-18 play an essential role in T cell and B cell activation [133]. This section looks at the function of NLRP3 activation in T and B cells within the context of autoimmune disorders (Fig. 4).

**Fig. 4:**
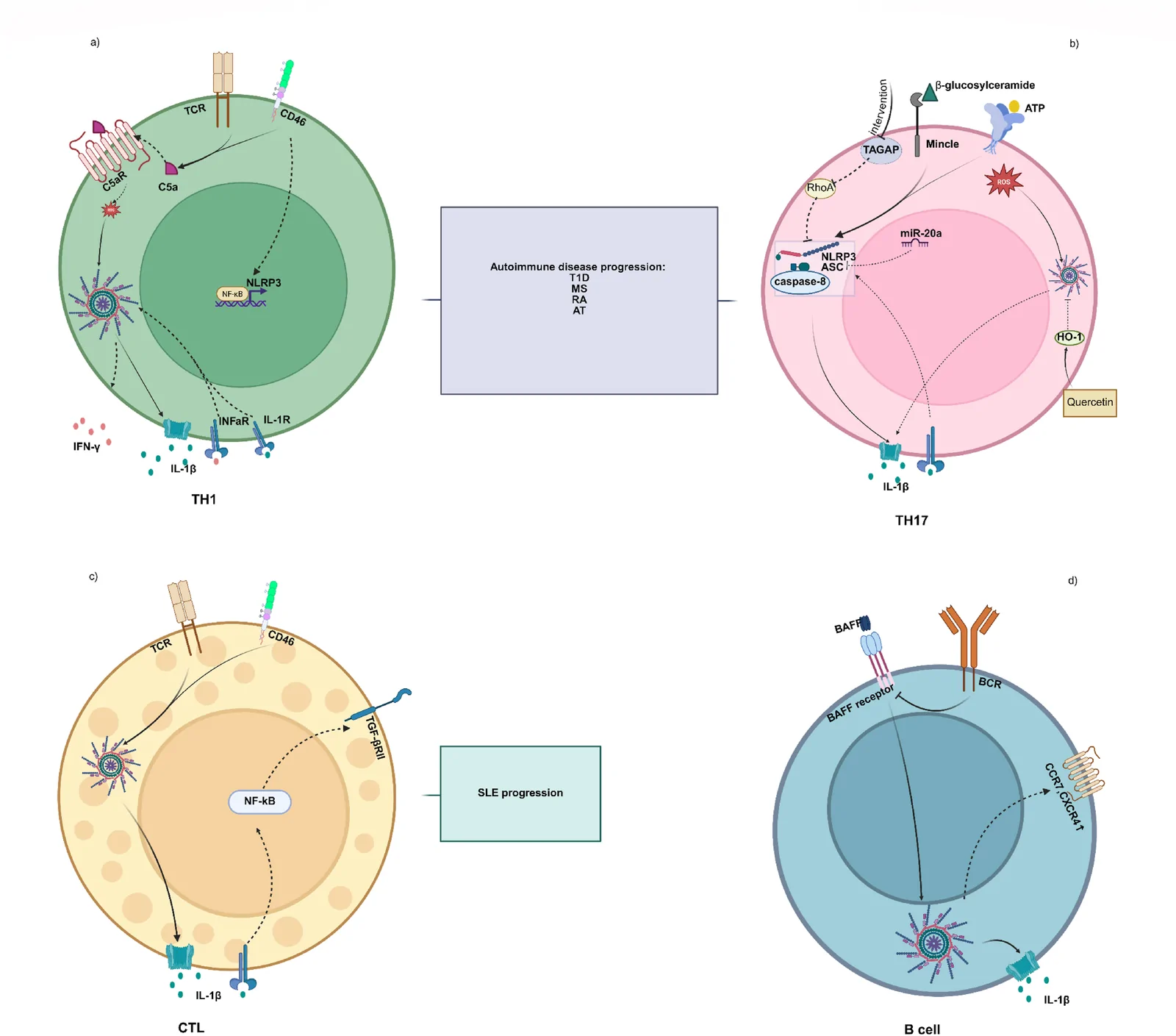
The role of NLRP3 inflammasome activation in adaptive immune cells in autoimmune disorders. a) CD46 and TCR signaling lead to intracellular C5a activation and NLRP3 inflammasome oligomerization. CD46 provides both priming and activating signals in Th1 cells. IL-1β and IFNγ enhance NLRP3 inflammasome activation in a positive feedback loop. b) Mincle signaling increases NLRP3, ASC and caspase-8 activation in Th17 cells. c) IL-1β promotes TGFBRII expression in an autocrine manner in CTLs. d) BCR activation inhibits NLRP3 inflammasome activation through BAFFR suppression. (AT: autoimmune thyroiditis؛ ATP: adenosine triphosphate؛ BAFFR: B cell-activating factor receptor؛ BCR: B-cell receptor؛ CCR7: C-C chemokine receptor type 7؛ CTL: cytotoxic T cell؛ CXCR-4: C-X-C chemokine receptor type 4؛ GBS: Guillain-Barré Syndrome؛ HO1: heme oxygenase 1؛ MS: Multiple sclerosis؛ RA: rheumatoid arthritis؛ RhoA: Ras homolog family member A؛ SLE: systemic lupus erythematosus؛ T1D: type 1 diabetes؛ TAGAP: T-cell activation Rho GTPase activating protein؛ TCR: T cell receptor؛ TGF-ΒRII: transforming growth factor beta receptor type 2؛ TH: T helper cell. Created in Biorender.com).

### 4.1 T helper cells (Th)

CD4^+^ T cells, which include subtypes such as Th1, Th2, Th17, follicular helper T (Tfh), and regulatory T (Treg) cells, are key components of the immune system [134]. Research indicates that functional NLRP3 assembles in human CD4^+^ T cells, leading to caspase-dependent IL-1β secretion. This, in turn, induces optimum IFNγ secretion and Th1 differentiation via an autocrine pathway. Assembly of NLRP3 requires T cell receptor-mediated intracellular C5a activation and stimulation of the C5 receptor, leading to ROS generation, which provides the required second signal for NLRP3 activation, whereas CD46 engagement increases NLRP3 gene transcription and serves as a priming signal [135]. In NOD mice, NLRP3 and pro-IL-1β gene expression is elevated in the pancreatic lymph nodes (PLNs). Additionally, caspase-positive macrophages are increased in the PLNs of diabetic mice. mtDNA significantly enhances IL-1β secretion and caspase-1 activation in wild-type macrophages. An increase in CD4^+^ T cells secreting IFNγ and IL-17, as well as CD8^+^ T cells secreting IL-17 and IFNγ, was observed in the PLNs of diabetic mice but not in NLRP3−/− mice [136]. Supporting these findings, in NLRP3-deficient NOD mice, CD69 and IFNγ expression in CD4^+^ T cells is reduced, and their proliferation in response to anti-CD3 stimulation is impaired. Additionally, APCs in NLRP3-deficient mice suppress the differentiation of naïve CD4^+^ T cells into Th1 cells. The lack of NLRP3 also leads to decreased expression of CCR5 and CXCR3 in CD4^+^ T cells and reduced CCL5 and CXCL10 levels in pancreatic islet cells, impairing CD4^+^ T cell chemotaxis [137].

Beyond its role in T1D, the NLRP3 inflammasome has been implicated in EAE. CD4^+^ T-cell migration to the CNS is impaired in Asc−/− and Nlrp3−/− mice. The NLRP3 inflammasome promotes EAE progression by enhancing the expression of chemotaxis-related molecules in both CD4^+^ T cells and APCs. CD4^+^ T cells require priming by NLRP3 inflammasome-sufficient APCs to acquire enhanced migratory capacity, whereas the migration defect is not T-cell intrinsic [138]. A further investigation indicated that the lack of NLRP3 diminishes the encephalitogenic responses of Th1 and Th17 cells, resulting in decreased inflammation and demyelination in the spinal cords of NLRP3−/− mice. Furthermore, lower IL-18 levels in NLRP3−/− mice reduced EAE severity as well as Th1 and Th17 cell cytokine production [139]. IL-1β also has a role in the survival, expansion, and function of Th17 cells. In detail, T cell receptor activation induces pro-IL-1β expression, while ATP stimulation leads to IL-1β secretion through the ASC-NLRP3-caspase-8-dependent pathway. The IL-1β receptor on Th17 cells further amplifies Th17 survival in an autocrine fashion, exacerbating CNS inflammation and causing EAE [140].

In Th cells, various pathways activate NLRP3. For instance, Mincle, a danger signal receptor commonly found in myeloid cells, is also expressed in Th17 cells. Recognition of β-glucosylceramide released by dying cells stimulates the ASC-NLRP3 pathway, inducing caspase-8-dependent IL-1β production by Th17 cells. IL-1β promotes Th17 proliferation in an autocrine manner and contributes to EAE progression [141]. Moreover, P2X7R expression is elevated in CD4^+^ T cells of patients with Guillain-Barré syndrome (GBS), an acute polyneuropathic inflammatory disorder driven by the immune system. Inhibition of P2X7R with brilliant blue G (BBG) suppresses the NLRP3 inflammasome and IL-1β at the protein level, improving experimental autoimmune neuritis (EAN) by diminishing Th1 and Th17 responses in rats immunized with the P0180–199 peptide [142]. In patients with RA, increased ROS generation in human CD4^+^ T cells promote NLRP3 inflammasome activation, accompanied by increased caspase-1 activation and IL-1β production. Treatment with the ROS inhibitor Mito TEMPO reduced caspase-1 activation and IL-1β production and impaired Th17 differentiation. These findings suggest that ROS-mediated NLRP3 inflammasome activation contributes to Th17 differentiation in RA. Therefore, NLRP3 represents a potential therapeutic target in RA [65]. Similarly, the P2X7R-NLRP3 pathway plays a role in the pathogenesis of CIA and T-cell differentiation. NLRP3 knockout mice demonstrate a decrease in Th17 cells and an elevation in Treg cells. There is a substantial reduction in miR-107 expression in CIA. The cytoplasmic long non-coding RNA (lncRNA) ENSMUST00000197208 sequesters miR-107, thereby promoting CD4^+^ T cell differentiation into Th17 cells via the P2X7R-NLRP3 pathway and elevating the Th17/Treg ratio. In ENSMUST00000197208 knockout (KO) mice, decreased Th17 differentiation results in reduced joint inflammation [143]. In line with this, serum analysis of patients with active RA shows that Protectin DX (PDX) levels are diminished. At the molecular level, PDX suppresses NLRP3 via miR-20a, modulating the Th17/Treg ratio and ameliorating CIA symptoms [144]. At the level of intracellular immune signaling regulation, T-cell-activation Rho GTPase-activating protein (TAGAP), which plays a role in Th17 differentiation, is implicated in various autoimmune disorders, including RA. Intervention with TAGAP in a rat model of CIA diminishes CD4^+^ T cell inflammatory responses and suppresses the expression of RhoA and NLRP3, thereby inhibiting Th17 differentiation and modulating the Th17/Treg balance [145].

Pharmacological interventions to suppress NLRP3 are also beneficial for reducing Th17 and Th1 numbers in autoimmune conditions. For instance, quercetin suppresses the NLRP3 inflammasome by enhancing the production of heme oxygenase 1 (HO1), a catabolic enzyme involved in anti-inflammatory responses. Quercetin decreases the Th17/Treg ratio and alleviates CIA symptoms in the mouse model [146].

Highlighting the significant role of autoantibodies and Tfh cells in SLE, NLRP3+ Tfh cells are present in normal B6 mice. These CD4+CXCR5+ Tfh cells secrete IL-1β upon activation of the NLRP3 inflammasome in response to immunization. Inhibition of the NLRP3 inflammasome with MCC950 prevented germinal center formation and reduced B-cell proliferation. MCC950 decreased spleen weight and inhibited the expression of IL-21, CD40L, pSTAT3, and B-cell lymphoma 6 (BCL6) in Tfh cells, ultimately reducing plasma cells and anti-dsDNA antibodies. In SLE patients, circulating NLRP3+ Tfh cell levels are elevated, and this population may represent a promising therapeutic target [147]. Together, accumulating evidence indicates that CD4^+^ T cells express an intrinsic NLRP3 inflammasome in autoimmune settings. Activation of this pathway through stimuli such as ROS and extracellular ATP promotes IL-1β production and enhances Th1 and Th17 inflammatory responses. These findings suggest that NLRP3 inflammasome activation within CD4^+^ T cells contribute to autoimmune pathology, including EAE and RA, through the regulation of T-cell differentiation and survival. NLRP3-mediated priming of naïve T cells by APCs can also increase the migratory capacity of T cells. However, the precise mechanisms and disease-specific roles of T-cell–intrinsic NLRP3 activation require more investigation to determine how NLRP3 activation shapes adaptive immune cell responses.

### 4.2 Cytotoxic T cell (CTL)

CD8^+^ CTLs are involved in autoimmune diseases [148], [149]. Little evidence exists for the role of the NLRP3 inflammasome in these cells. In isolated human cell culture, Arbore et al. reported that CD8^+^ T cells express NLRP3. TCR activation, especially when accompanied by complement receptor CD46, increases NLRP3 expression in CTLs. Unlike CD4^+^ T cells, inhibition of the NLRP3 inflammasome with MCC950 does not reduce caspase-1 production in CD8^+^ T cells [150]. In the context of SLE, another study has demonstrated that the NLRP3 inflammasome is activated in CD8^+^CD69^+^CD103+ TRM cells from a mouse model of lupus with LN, as well as in human-like CD8^+^CD103^+^ T cells in vitro. Activation of the NLRP3 inflammasome in these cells plays a role in the pathogenesis of LN. In this mouse model, systemic pharmacological inhibition of NLRP3 with MCC950 reduces the infiltration and activity of these cells in the kidney and improves renal injury. In vitro experiments suggested that autocrine IL-1β increases TGF-βRII gene expression in CD8^+^ T cells through the NF-κB pathway and contributes to TGF-β-dependent differentiation of these cells [151]. Further research is needed to understand whether these effects reflect a direct cell-intrinsic role of NLRP3 in CD8^+^ T cells or indirect systemic modulation.

### 4.3 B cells

B cells play an important role in the pathogenesis of autoimmune diseases by producing autoantibodies, interacting with T cells, and presenting antigens [152]. Research shows that the NLRP3 inflammasome is expressed in B cells and activated in response to pathogens [153], [154]. There is evidence showing that NLRP3 interacts with IRF4 in the nucleus of B cells; in NLRP3−/− mice, IRF4 expression is upregulated, resulting in altered allelic exclusion and light-chain rearrangement. NLRP3−/− mice also showed increased expression of the chemokine receptors CXCR4 and CCR7, which was associated with altered B-cell homing and positioning. NLRP3 deficiency altered the distribution of peripheral B-cell subsets, with an increased proportion of follicular B cells and a >50% reduction in marginal zone B cells in the spleen, accompanied by expansion of B-1a cells in the peritoneal cavity. Notably, serum from global NLRP3−/− mice showed higher levels of polyreactive natural antibody production compared with WT mice [155], though global knockout phenotypes may also reflect systemic microenvironmental changes alongside cell-intrinsic effects.

The B cell-activating factor (BAFF) receptor activation constitutes an additional pathway that activates the NLRP3 inflammasome in B cells. Lim et al. discovered that BAFF enhances IL-1β production and activates caspase-1 by binding to its receptor. Upon activation of the NLRP3 inflammasome, B cells release IL-1β and undergo pyroptosis. Stimulation of the B-cell receptor (BCR) inhibits BAFF receptor signaling and obstructs the activation of the NLRP3 inflammasome [156]. In isolated human circulating B cells, NLRP3 activation by two stimuli has distinct consequences. Fungal β-glucan activates NLRP3 and caspase-1, leading to IL-1β release. Mechanistically, recognition of β-glucan by Dectin-1 activates SYK and the downstream NF-κB/AP-1 pathway, ultimately promoting IL-1β production. However, NLRP3 activation by CpG does not follow the same downstream pathway. CpG stimulates TLR9, which activates NLRP3 and the mTOR-related pathway, resulting in IgM secretion. IL-1β and IgM secretion are higher in memory B cells compared with naïve B cells [157]. This evidence indicates that NLRP3 has a functional role in B cells; however, the exact cell-intrinsic role of NLRP3 inflammasome activation in B cells in autoimmune diseases remains unknown and requires further investigation.

## 5 Clinical trials and NLRP3 therapeutic potentials

Analysis of renal biopsies from patients with LN demonstrated that NLRP3 expression was higher in tubular cells, particularly in class IV LN, and positively correlated with the activity index score [158]. In line with this, kidney tissue biopsies from LN patients and MRL/lpr mice show NLRP3 activation and caspase-1/GSDMD-mediated pyroptosis [159]. An analytical study indicated that PYCARD and IL1B expression were higher in patients with SLE and could serve as diagnostic markers [160]. Thus, targeting the NLRP3 inflammasome activation pathway may be a promising strategy in autoimmune diseases.

Various small molecules have been designed to inhibit the NLRP3 inflammasome. Limited clinical trials have been undertaken in this context to date, as shown in Table 1. NodThera announced that its investigational small-molecule NLRP3 inhibitor, NT-0796, which has CNS penetration, completed a Phase 1 clinical trial for neuroinflammatory diseases, with no drug-related liver toxicity detected; however, more investigation is needed to confirm its clinical efficacy and long-term safety [161], [162]. In another clinical trial, fenofibrate, a medication utilized for hypercholesterolemia and hypertriglyceridemia, has been evaluated for a novel application. This medication has been utilized to modulate the mammalian target of rapamycin (mTOR)/NLRP3 pathway and suppress the NF-κB pathway in patients with ulcerative colitis, and the phase 2 clinical trial was completed [163]. A comparable phase 2 clinical trial is utilizing the medication atorvastatin. Atorvastatin is a lipid-lowering medication possessing anti-inflammatory and antioxidant characteristics. This trial uses it to modulate the mTOR/NLRP3 pathway in patients with ulcerative colitis [164]. On the other hand, Selnoflast (RO7486967), a selective NLRP3 inhibitor, was evaluated in a study involving 19 patients with UC over 7 days. Selnoflast was well tolerated with drug exposure exceeding the predicted IL-1β IC90; however, tissue pharmacodynamic effects were insufficient to support continued development in ulcerative colitis and there was no difference between the Selnoflast- and placebo-treated groups in IL-1-related gene expression in sigmoid colon tissue or in stool biomarkers [165], showing that adequate drug exposure, even in the target tissue, does not ensure downstream pathway suppression or clinical benefit.

**Table 1:** Clinical trials of NLRP3 and downstream pathway inhibition in autoimmune disorders.

Inhibitor	Type of Therapeutic Intervention	Disease	Clinical Trials Phase	Mechanism of Inhibitor	Findings	References	NCT Number
NT0796	Direct NLRP3 inhibitor	Neuroinflammatory disease	Phase 1 (completed)	Blocking of NLRP3 assembly	No liver toxicity detected	[161]	
Fenofibrate	Indirect modulator/lipid-lowering medication	Ulcerative colitis	Phase 2 (completed)	Modulates mTOR/NLRP3 and suppresses NF-κB pathway	Enhancing therapeutic results, patient well-being, and regulating the inflammatory response	[163]	NCT05781698
Atorvastatin	Indirect modulator/lipid-lowering medication	Ulcerative colitis	Phase 2	Targets mTOR/NLRP3 pathway	Enhancing therapeutic results, patient well-being, and regulating the inflammatory response	[164]	NCT05567068
Selnoflast (RO7486967)	Direct NLRP3 inhibitor	Ulcerative colitis	Phase 1b (completed)	Blocking of NLRP3 assembly	Well tolerated; no clear tissue pharmacodynamic effect supporting continued UC development	[165]	
Curcumin	Natural compound with an indirect NLRP3-inhibitory effect	Pediatric lupus nephritis	Phase 2 (completed)	Inhibits NLRP3 upstream activation pathways	Awaiting further results		NCT05714670
Disulfiram	downstream inflammasome-effector inhibitors/aldehyde dehydrogenase inhibitor	Rheumatoid Arthritis	Phase 2	Inhibits GSDMD-mediated signaling and pyroptosis	Awaiting further results		NCT07534332

mTOR: mechanistic target of rapamycin; NF-κB: nuclear factor kappa-light-chain-enhancer of activated B cells; NLRP3: NOD-, LRR- and pyrin domain-containing protein 3; GSDMD: gasdermin D.

Although clinical trials using NLRP3 inflammasome inhibitors in treating autoimmune diseases are in their early phases, various investigations have been performed in mice models (Table 2). NLRP3 inflammasome small molecule inhibitors have been used in various studies. For example, the administration of MCC950 in the C57BL/6 mice model of EAE enhances outcomes and mitigates pathological damage in the spinal cord. Moreover, suppressing the NLRP3 inflammasome diminishes glial cell activation and inhibits microglial polarization towards the M1 phenotype [86]. The inhibitory effect of MCC950 was also studied in mice with EAT. MCC950 diminishes the infiltration of CD4^+^ and CD8^+^ cells in the thyroid by inhibiting the NLRP3 inflammasome within the thyroid parenchyma. It modulates the Th1/Th17/Treg ratio by enhancing FoxP3 expression while reducing T-bet and RORγt expression. Additionally, MCC950 markedly attenuates Th1 and Th17 responses and facilitates the activation of Treg cells [166]. RRX-001 is an anticancer agent in terminated Phase 3 clinical trials for small-cell lung cancer [167]. RRX-001 inhibits the interaction between NLRP3 and NEK7 by binding to cysteine 409 in the NACHT domain of NLRP3. The anti-inflammatory properties of RRX-001 have been investigated in mice with EAE and DSS-induced colitis. RRX-001 treatment reduced serum levels in mice with DSS-induced colitis. In EAE mice, the infiltration of CD11b^−^, CD11b^+^, CD8^+^, and CD4^+^ cells into the CNS were reduced. RRX-001 ameliorated signs of disease in both mice models by decreasing inflammation [168]. Another investigation found that administering OLT1177 (Dapansutrile), an NLRP3 inflammasome inhibitor, improved EAE. OLT1177 decreased the number of immune cells infiltrating the spinal cord and significantly alleviated inflammation [169]. OLT1177 suppresses NLRP3 activation downstream of P2X7R. The Phase 1 clinical trial has been successfully completed in healthy humans [170]. 1,2,4-Trimethoxybenzene (1,2,4-TTB), a specific inhibitor of the NLRP3 inflammasome, inhibits NLRP3 activation by blocking ASC oligomerization. In the EAE model, the administration of 1,2,4-TTB lowers the mRNA levels of IFN-γ and IL-17A, inhibiting pathogenic T-cell activation [171]. Manoalide is an NLRP3 inflammasome inhibitor that prevents its activation following K+ and Cl-efflux and mitochondrial dysfunction. Manoalide inhibits its activation by covalently attaching to Lys 377 in the NACHT domain of the NLRP3 protein. The administration of manoalide to EAE mice decreases the infiltration of CD11b^−^, CD11b^+^, CD4^+^, and CD8^+^ cells in the brain. The production of inflammatory cytokines is reduced, leading to an amelioration of EAE symptoms [67].

**Table 2:** Preclinical studies targeting the NLRP3 inflammasome pathway in autoimmune diseases.

Inhibitor	Type of intervention	Disease model	Findings	Mechanism of NLRP3 inhibition	References
MCC950	Direct NLRP3 inhibitor	Experimental autoimmune encephalomyelitis (EAE)	Reduces spinal cord damage, suppresses glial activation, and inhibits M1 microglial polarization	Blocks ATPase activity of NLRP3, preventing its activation	[86]
		Experimental Autoimmune Thyroiditis (EAT)	Reduces CD4^+^ T cell and CD8^+^ T cell infiltration, modulates Th1/Th17/Treg ratio	Blocks ATPase activity of NLRP3, preventing its activation	[166]
RRX-001	Direct NLRP3 inhibitor	EAE & DSS-induced colitis	Reduces inflammation and immune cell infiltration	Inhibits NLRP3-NEK7 interaction by binding to cysteine 409 in the NACHT domain	[168]
OLT1177 (Dapansutrile)	Direct NLRP3 inhibitor	EAE	Decreases immune cell infiltration and spinal cord inflammation	Suppresses NLRP3 activation downstream of P2X7R	[169]
1,2,4-Trimethoxybenzene (1,2,4-TTB)	Direct NLRP3 inhibitor	EAE	Lowers IFN-γ and IL-17A mRNA levels, inhibits pathogenic T-cell activation	Blocks ASC oligomerization to inhibit NLRP3 activation	[171]
Manoalide	Direct NLRP3 inhibitor	EAE	Reduces CD11b-, CD11b+, CD4^+^, and CD8^+^ cell infiltration, decreases cytokine production	Covalently binds to Lys 377 in the NACHT domain to prevent NLRP3 activation	[67]
miRNA-20a	Indirect NLRP3 modulator	Adjuvant-induced arthritis (AA)	Suppresses NLRP3 inflammasome by targeting TXNIP mRNA	Binds to the 3′UTR of TXNIP mRNA, reducing NLRP3 activation under oxidative stress	[172]
Let-7f-5p	NLRP3 expression regulator	SLE	Reduces NLRP3 levels and inflammatory cytokines in MRL/lpr mice	Directly targets NLRP3 mRNA, decreasing its expression	[173]
Exosomal miR-223	NLRP3 expression regulator	RA	Suppresses NLRP3 inflammasome in macrophages, reduces cytokine release	Downregulation of NLRP3 expression	[99]
Tranilast (TR)	Direct NLRP3 inhibitor	Arthritis	Reduces IL-1β production in synovial fluid cells	Directly binds to NLRP3, preventing its oligomerization and activation	[174]

ASC: Apoptosis-associated speck-like protein containing a CARD; ATPase: Adenosine triphosphatase; DSS-induced colitis: Dextran sulfate sodium-induced colitis; IFN-γ: Interferon-gamma; let-7f-5p: Lethal-7f-5p microRNA; MCC950: A selective NLRP3 inflammasome inhibitor; miRNA-20a: MicroRNA-20a; MRL/lpr: Murphy Roths Large/lymphoproliferation; NACHT: NAIP, CIITA, HET-E, and TP1 domain; NEK7: NIMA-related kinase 7; NLRP3: NOD-like receptor family pyrin domain-containing 3; P2XR7: Purinergic receptor P2X ligand-gated ion channel 7; RA: Rheumatoid arthritis; RRX-001: A small-molecule NLRP3 inhibitor; SLE: Systemic lupus erythematosus; TXNIP: Thioredoxin-interacting protein; UTR: Untranslated region.

Besides small molecules, miRNAs that suppress the NLRP3 inflammasome have been investigated. miRNA-20a inhibits the activation of the NLRP3 inflammasome in adjuvant-induced arthritis (AA) fibroblast-like synoviocytes (FLSs) by binding to the 3′UTR of TXNIP mRNA, which is an activator of the NLRP3 inflammasome under oxidative stress conditions [172]. Let-7f-5p directly targets NLRP3. Serum analysis of SLE patients indicates a significant reduction in let-7f-5p levels. Elevated Let-7f-5p expression lowers mRNA levels of IL-1β, ASC, and caspase-1. The intravenous administration of SLE-BMSCs with lenti-let-7f-5p into MRL/lpr mice enhances Let-7f-5p expression and diminishes NLRP3 levels. Serum concentrations of the cytokines IFNγ, IL-6, IL-12, IL-13, IL-18, and IL-1β are reduced, signifying a decreased inflammation in MRL/lpr mice with NLRP3 modulation [173]. Utilizing exosomes released by BMSCs represents an alternative strategy to suppress the NLRP3 inflammasome. Exosomes containing miR-223 may be effective in treating RA by suppressing the NLRP3 inflammasome in macrophages and decreasing the release of inflammatory cytokines [99]. Another approach involves repurposing drugs with novel targets. Tranilast (TR) is an antiallergic agent that has been investigated for its inhibitory effect on NLRP3. TR inhibits oligomerization by directly binding to NLRP3, thereby blocking the activation of the NLRP3 inflammasome complex in both murine and human cells. Research on synovial fluid from arthritis patients indicates that the incubation of synovial fluid cells with TR reduces caspase-1 activation and IL-1β production in a dose-dependent manner [174]. Taken together, preclinical studies demonstrate that NLRP3 inhibition improves autoimmune disease outcomes. However, clinical evidence supporting its effectiveness in treating patients with autoimmune disorders remains limited.

It is noteworthy that, beyond the challenge of identifying a clinically safe and effective NLRP3 inhibitor, the potential long-term adverse effects of suppressing inflammasome-mediated host-defense responses to pathogens are another concern that should be considered in translational therapy. For instance, the NLRP3 inflammasome, particularly IL-18, may have a protective role during HIV infection [175]. However, the beneficial or detrimental effects of NLRP3 inflammasome activation during infection are highly context- and stage-dependent. For example, during influenza A virus infection, NLRP3 activation in the early phase contributes to viral clearance, whereas later activation may promote NLRP3-mediated inflammation, leading to increased lung cellular infiltration and disease severity [176]. Notably, genetic variation in NLRP3 among humans may represent another challenge to translational efficacy. A meta-analysis of NLRP3 polymorphisms demonstrated that the association of certain polymorphisms with autoimmune disease susceptibility varies according to both disease type and ethnicity. Specifically, rs10754558 was associated with susceptibility to some autoimmune diseases but not others, and this association was observed in Latin American populations but not in European, Arabian, or Asian populations [177]. To address why NLRP3 inhibition succeeds in preclinical autoimmune models but is difficult to translate clinically, it is informative to consider other NLRP3-driven disorders. For instance, in cardiac disease, MCC950 reduced arrhythmias and fibrosis in animal models, but NLRP3 activation has not been well quantified in-patient tissues, and prospective studies of NLRP3 inhibitors in this setting are lacking [178]. Also, MCC950 showed strong preclinical activity but caused hepatotoxicity and was discontinued in a Phase II rheumatoid arthritis trial. This shows that potent pathway inhibition does not guarantee clinical success, and that safety and pharmacokinetic profiling must be evaluated alongside pharmacodynamics. A candidate compound must be safe, well tolerated, and able to reach the diseased tissue at pharmacologically active concentrations (e.g., crossing the blood-brain barrier in MS). Patient-related factors, including age and genetic background (e.g., NLRP3 polymorphisms), must also be considered. Finally, tissue biopsies, where feasible, would allow direct assessment of target engagement rather than reliance on indirect systemic markers such as CRP and IL-6 [179]. Collectively, the therapeutic potential of NLRP3 inhibition in humans still needs further investigation to address the complexity and context- and cell-dependent effects of NLRP3 in autoimmune diseases.

## 6 Conclusion

Although NLRP3 is primarily recognized as an innate immune receptor activated by DAMPs and PAMPs, it is also expressed in adaptive immune cells. Understanding its role in immune activation in the context of autoimmune disease may contribute to the design of new therapeutic strategies. As described, NLRP3 inflammasome activation requires two signals: a priming signal, mediated by TLRs, TNFR, and IL-1R through NF-κB activation, which upregulates the expression of NLRP3, pro-IL-1β, and pro-IL-18, and an activating signal triggered by factors such as K+ efflux, ROS, extracellular ATP, lysosomal rupture, and mitochondrial dysfunction, leading to NLRP3 oligomerization and caspase-1 activation, followed by the maturation and release of IL-1β and IL-18, as well as GSDMD-mediated pyroptosis. These cytokines enhance immune responses in an autocrine and paracrine manner, fostering inflammation and activating immune cells. Unlike its well-characterized role in innate immune cells, evidence shows functional NLRP3 activation within lymphocytes; however, the precise function of NLRP3 in adaptive immune cells, particularly B cells and cytotoxic T lymphocytes, in the context of autoimmunity requires further investigation. NLRP3 inflammasome inhibitors, which hamper polarization toward the inflammatory macrophage phenotype and promote tolDC generation, have shown efficacy in reducing inflammation. The involvement of the NLRP3 inflammasome in the pathophysiology of autoimmune diseases makes it an attractive therapeutic target (a cross-disease comparison is summarized in Table 3).

**Table 3:** Cross-disease comparison of NLRP3 inflammasome activation in major autoimmune disorders.

Autoimmune disease	Predominant NLRP3-involved cell(s)	Trigger/pathway of NLRP3 activation	Inhibitor/therapeutic intervention
EAE	Microglia/macrophages, neutrophils, Th1/Th17 cells, meningeal mast cells, MDSCs	ROS/NF-κB/NLRP3 Pyroptosis K+/Cl- efflux NETs	MCC950 RRX-001 OLT1177 1,2,4-TTB Manoalide
SLE	Neutrophils (NETosis), macrophages, Tfh cells, CD8^+^ TRM cells	Anti-dsDNA (mitochondrial ROS + TLR4–NF-κB), NETs + LL-37, S1P/S1PR	Let-7f-5p (decreased NLRP3 in MRL/lpr model)
RA	Synovial macrophages (CD14^+^), CD4^+^ T cells, mast cells, MDSCs	ACPA (via CD147–ITGB1–Akt–NF-κB), ROS in CD4^+^ T cells, TNFα/RelA (decreased miR-30a)	Quercetin MCC950
IBD (colitis)	Colonic macrophage	Loss of IL-10 (in IL-10−/− mice), VDAC1 overexpression, OTUD6A (inhibits NLRP3 proteasomal degradation)	Glyburide RRX-001

ACPA: anti-citrullinated protein antibody; EAE: experimental autoimmune encephalomyelitis; IBD: inflammatory bowel disease; MDSC: myeloid-derived suppressor cell; MRL/lpr: Murphy Roths Large/lymphoproliferation; NETs: neutrophil extracellular traps; RA: rheumatoid arthritis; ROS: reactive oxygen species; SLE: systemic lupus erythematosus; VDAC1: voltage-dependent anion channel 1; OTUD6A: ovarian tumor deubiquitinase 6A

Small-molecule inhibitors such as MCC950 have demonstrated effectiveness in animal models of EAE, RA, autoimmune thyroiditis (AIT), and SLE by alleviating inflammation, but no direct NLRP3 inhibitor has yet received regulatory approval for the treatment of autoimmune diseases. miRNAs effectively inhibit NLRP3 and modulate immune responses. For example, miR-223-3p directly interacts with NLRP3, downregulating caspase-1 activation and IL-1β release. However, several challenges limit the translational efficiency of NLRP3-targeted therapies. Beyond the potential adverse effects that inhibitors such as MCC950 may cause in humans, long-term inhibition of NLRP3 may weaken immune defense against infections. Considering the cell-specific role of NLRP3 activation and the stage of disease, NLRP3 inhibition may have distinct consequences. Further research is needed to assess the long-term efficacy and safety of these inhibitors in human autoimmune diseases.

## CRediT authorship contribution statement

**Aysan Asefpour:** Writing – review & editing, Writing – original draft, Visualization, Investigation. **Shiva Alipour:** Writing – review & editing, Conceptualization. **Sepideh Sohrabi:** Writing – review & editing. **Yasser Bagheri:** Writing – review & editing, Supervision. **Leili Aghebati-Maleki:** Writing – review & editing, Validation, Supervision, Conceptualization.

## Consent for publication

All authors read and approved the final manuscript for publication.

## Clinical trial number

not applicable.

## Funding

This study was supported by Research Vice- Chancellor at 10.13039/501100004366Tabriz University of Medical Sciences, Iran [Grant No. 74289].

## Declaration of competing interests

The authors declare the following financial interests/personal relationships which may be considered as potential competing interests: Leili Aghebati-Maleki reports financial support was provided by Research Vice- Chancellor at Tabriz University of Medical Sciences, Iran. If there are other authors, they declare that they have no known competing financial interests or personal relationships that could have appeared to influence the work reported in this paper.

## Data Availability

No data was used for the research described in the article.

## Abbreviations

3′UTR: 3′-untranslated region
AA: Adjuvant-induced arthritis
ACPA: Anti-citrullinated protein antibody/antibodies
AIH: Autoimmune hepatitis
AIM2: Absent in melanoma 2
AIT: Autoimmune thyroiditis
ALRs: AIM2-like receptors
APCs: Antigen-presenting cells
ASC: Apoptosis-associated speck-like protein containing a CARD
ATPase: Adenosine triphosphatase
BAFF: B cell-activating factor
BBG: Brilliant blue G
BCL6: B-cell lymphoma 6
BCR: B-cell receptor
BIR: Baculovirus inhibitor (of apoptosis) repeat (domain)
BMDMs: Bone marrow-derived macrophages
BMSCs: Bone marrow mesenchymal stem cells
C-CIA: Citrullinated collagen-induced arthritis
CARD: Caspase (activation and) recruitment domain
cDCs: Conventional dendritic cells
CIA: Collagen-induced arthritis
CLIC1/4: Chloride intracellular channel proteins 1 and 4
CPZ: Cuprizone
CTL: Cytotoxic T (lymphocyte/cell)
DAMPs: Damage-associated molecular patterns
DCs: Dendritic cells
DSS: Dextran sulfate sodium
dsDNA: Double-stranded DNA
EAE: Experimental autoimmune encephalomyelitis
EAM: Experimental autoimmune myocarditis
EAN: Experimental autoimmune neuritis
EAT: Experimental autoimmune thyroiditis
ERK1/2: Extracellular signal-regulated kinase 1/2
FADD: Fas-associated protein with death domain
FIIND: Function-to-find domain
FOXP3: Forkhead Box P3
GM-CSF: Granulocyte-macrophage colony-stimulating factor
GSDMD: Gasdermin D
HK2: Hexokinase 2
HO1: Heme oxygenase 1
HT: Hashimoto's thyroiditis
hucMSC: Human umbilical cord mesenchymal stem cell
hUCB-MSCs: Human umbilical cord blood-mesenchymal stem cells
IBD: Inflammatory bowel disease
IFN-β: Interferon-beta
IFNAR: Type I interferon receptor
IL-1β: Interleukin-1β
IP3R: Inositol trisphosphate receptor
ITGB1: Integrin beta 1
JAK2: Janus kinase 2
LN: Lupus nephritis
lncRNA: Long non-coding RNA
LRR(s): Leucine-rich repeat(s)
MAVS: Mitochondrial antiviral-signaling protein
MDSCs: Myeloid-derived suppressor cells
MHC: Major histocompatibility complex
MMP-9: Matrix metalloproteinase-9
MOG: Myelin oligodendrocyte glycoprotein
MRL/lpr: Murphy Roths Large/lymphoproliferation
MS: Multiple sclerosis
mTOR: Mammalian target of rapamycin
NACHT: Central nucleotide-binding domain
NEAT1: Nuclear paraspeckle assembly transcript 1
NEK7: NIMA-related kinase 7
NETs: Neutrophil extracellular traps
NF-κB: Nuclear factor kappa B
NK: Natural killer (cells)
NLRP3: NOD-, LRR-, and pyrin domain-containing protein 3
NLRs: Nucleotide-binding and oligomerization domain-like receptors/NOD-like receptors
P2RX7: Purinergic receptor P2X ligand-gated ion channel 7
PAD4: Peptidyl arginine deiminase 4
PAMPs: Pathogen-associated molecular patterns
PBMCs: Peripheral blood mononuclear cells
PDX: Protectin DX
PI3K: Phosphoinositide 3-kinase
PKB: Protein kinase B (Akt)
PLNs: Pancreatic lymph nodes
PMN: Polymorphonuclear
PRR(s): Pattern recognition receptor(s)
PYD: Pyrin domain
RA: Rheumatoid arthritis
ROS: Reactive oxygen species
S1P: Sphingosine-1-phosphate
S1PR: S1P receptor
SAA: Serum amyloid A
SLE: Systemic lupus erythematosus
SS: Sjögren's syndrome
STAT: Signal transducer and activator of transcription
T1D: Type 1 diabetes
TAGAP: T-cell-activation Rho GTPase-activating protein
TCR: T cell receptor
Tfh: T follicular helper (cells)
TGF-β: Transforming growth factor beta
Th1/2/17: T helper 1/2/17
TIM4: T-cell immunoglobulin and mucin domain-containing 4
TLR(s): Toll-like receptor(s)
TNF-α: Tumor necrosis factor alpha
Treg: Regulatory T (cell)
TRPM2: Transient receptor potential melastatin 2
TRPV1: Transient receptor potential vanilloid type 1
TWIK2: Two-pore domain weak inwardly rectifying K+ channel 2
TXNIP: Thioredoxin-interacting protein
UC: Ulcerative colitis
VDAC: Voltage-dependent anion channel
